# Effects of Lower Limb-Focused Low-Intensity Resistance Exercise Using Slow Movements on Locomotive Syndrome in Patients with Type 2 Diabetes Mellitus

**DOI:** 10.3390/medicina61101875

**Published:** 2025-10-19

**Authors:** Toru Morihara, Kazufumi Hisamoto, Naoki Okubo, Hideki Fukushima, Tomoyuki Matsui, Machiko Hiramoto, Masahide Hamaguchi, Hiroshi Okada, Takaaki Matsui, Dan Imai, Michiaki Fukui, Kenji Takahashi

**Affiliations:** 1 Marutamachi Rehabilitation Clinic, 12 Kurumazaka-cho Nishinokyo Nakagyo-ku, Kyoto 604-8405, Japan; toru4271@koto.kpu-m.ac.jp (T.M.); fukushima-hideki@rakuwa.or.jp (H.F.); matsui.tomoyuki.sports.reha@gmail.com (T.M.); hiramoto-machiko@rakuwa.or.jp (M.H.); 2 Department of Orthopaedics, Graduate School of Medical Science, Kyoto Prefectural University of Medicine, Kawaramachi-Hirokoji, Kamigyo-ku, Kyoto 602-8566, Japan; hisamoto@koto.kpu-m.ac.jp (K.H.); kenji-am@koto.kpu-m.ac.jp (K.T.); 3 Department of Endocrinology and Metabolism, Graduate School of Medical Science, Kyoto Prefectural University of Medicine, Kawaramachi-Hirokoji, Kamigyo-ku, Kyoto 602-8566, Japan; mhama@koto.kpu-m.ac.jp (M.H.); conti@koto.kpu-m.ac.jp (H.O.); tmastui@koto.kpu-m.ac.jp (T.M.); dimai@koto.kpu-m.ac.jp (D.I.); michiaki@koto.kpu-m.ac.jp (M.F.)

**Keywords:** diabetes mellitus, low-intensity resistance exercise, slow exercise, locomotive syndrome

## Abstract

*Background and Objectives*: Type 2 diabetes mellitus (T2DM) is a major public health issue worldwide that leads to reductions in skeletal muscle mass and lower-limb function, thereby increasing the risk of locomotive syndrome (LS), a mobility-limiting condition. This study investigated the effects of a 5-month, lower limb-focused, low-intensity resistance exercise program using slow movements (slow exercise) on LS in patients with T2DM. *Materials and Methods*: Nineteen patients with T2DM (69.3 ± 7.3 years, 10 men and 9 women) performed slow exercises three times per week for 5 months. The program consisted of bodyweight and machine-based exercises with a load of 30–50% one-repetition maximum using slow concentric–isometric–eccentric phases. Assessments included HbA1c, LS stage distribution (non-LS, LS-1, LS-2, LS-3), LS risk tests (two-step, stand-up, and GLFS-25), five-time sit-to-stand test, four-meter gait speed, and skeletal muscle mass index (SMI) and phase angle (PhA) by bioelectrical impedance analysis. Statistical comparisons were performed using repeated one-way ANOVA with Tukey’s post hoc test and Cochran’s Q test. *Results*: HbA1c decreased from 7.5 ± 0.7% to 7.2 ± 0.8% (*p* < 0.05). LS stage distribution improved significantly (stage 3: 4 to 0; non-LS: 1 to 5; each *p* < 0.05). The two-step test and GLFS-25 improved (1.17 ± 0.15 to 1.27 ± 0.17; 14.6 ± 13.1 to 7.6 ± 6.3; each *p* < 0.05). Five-time sit-to-stand time improved from 9.28 ± 2.53 s to 7.73 ± 1.54 s, and four-meter gait speed improved from 3.58 ± 0.95 s to 3.07 ± 0.55 s (each *p* < 0.05). SMI and PhA increased (6.82 ± 1.00 to 6.95 ± 0.92 kg/m^2^; 4.35 ± 0.67 to 4.56 ± 0.78 degrees; each *p* < 0.05). *Conclusions*: A 5-month slow exercise program significantly improved LS severity, muscle quantity and quality, and lower-limb function in patients with T2DM. Slow exercise may be a safe and effective intervention to improve mobility and maintain independence in this population.

## 1. Introduction

Globally, many countries have entered a super-aged society, accompanied by an increase in the number of elderly people requiring support [1]. Locomotive syndrome (LS), a concept proposed by the Japanese Orthopaedic Association in 2007, refers to a condition characterized by impaired mobility due to musculoskeletal disorders, which increases the risk of falls and fractures, ultimately leading to nursing care [2]. LS is assessed using the LS risk tests: the two-step test, the stand-up test, and the 25-question Geriatric Locomotive Function Scale (GLFS-25). Detailed procedures are provided in the Section 2; briefly, the two-step test measures the maximum distance covered in two consecutive steps, representing horizontal mobility performance. The stand-up test evaluates the capacity to rise from seats of different heights on one or both legs, reflecting vertical mobility. GLFS-25 is a self-administered questionnaire that assesses mobility-related difficulties in daily life. Based on these three assessments, LS severity is classified into non-LS and stages 1 to 3, with stage 3 indicating the most severe condition [3]. Yoshimura et al. have reported that LS 3 is associated with poor prognosis, including increased mortality [4]. Therefore, the prevention and improvement of LS are directly related to the maintenance of quality of life (QOL) and the extension of healthy life expectancy [5]. In Japan, the ROAD (Research on Osteoarthritis/Osteoporosis Against Disability) study, which included 1575 participants with a mean age of 65.6 years, reported that 41.3% were classified as stage 1, 14.9% as stage 2, and 11.6% as stage 3 according to the LS diagnostic criteria [4].

Type 2 diabetes mellitus (T2DM) is a major public health issue that leads to reductions in skeletal muscle mass and lower limb function [6], thereby increasing the risk of LS [7]. The global prevalence of diabetes among adults aged 20 to 79 years was estimated to be 10.5% (536.6 million people) in 2021 and is projected to rise to 12.2% (783.2 million) by 2045 [8]. Skeletal muscle is essential not only for motor function but also for glucose regulation via insulin-mediated glucose uptake [9]. In T2DM, persistent hyperglycemia causes inflammation in skeletal muscle, inhibits protein synthesis, and promotes protein degradation. Specifically, hyperglycemia impairs insulin signaling through the PI3K–AKT–mTOR pathway, thereby reducing muscle protein synthesis [10]. Simultaneously, it activates catabolic pathways such as ubiquitin–proteasome and autophagy-lysosome systems, accelerating muscle protein degradation [10]. Compared with non-diabetic individuals, older adults with T2DM have been reported to exhibit greater lower-limb muscle weakness, which further increases the risk of falls, reduces daily functional capacity, and negatively affects QOL [11]. Therefore, reducing the severity of LS in T2DM patients may also contribute to improving muscle metabolic function [12,13,14].

Physical activity and exercise are recommended to improve overall health and glycemic control in patients with T2DM [15,16]. In particular, resistance training is one of the most effective interventions for improving muscle mass, strength, and mobility in older adults [17,18]. High-load resistance training (approximately 85% of one-repetition maximum [1RM]) is effective in preventing physical decline [19], but it can cause excessive stress on the joints and circulatory system [20]. Therefore, in elderly patients with T2DM who have reduced exercise tolerance, high-load resistance training often causes joint pain and exertional dyspnea [21,22], leading to decreased adherence to exercise therapy. On the other hand, Watanabe et al. reported that low-intensity resistance training with a load of 30–50% 1RM using slow movement significantly improved muscle mass and strength in the elderly with no adverse events [23,24]. However, most studies on slow exercise have focused on healthy older adults, and a few studies have investigated its effects in patients with T2DM [25].

Given its potential to improve muscle mass and lower limb function without placing excessive stress on the joints or cardiopulmonary system, we hypothesized that slow exercise could serve as a safe and effective exercise intervention to reduce the severity of LS in patients with T2DM. To clarify this hypothesis, we investigated the effects of a 5-month lower limb-focused slow exercise program on LS in patients with T2DM.

## 2. Materials and Methods

### 2.1. Participants

The investigation was conducted between August 2022 and June 2024. Nineteen patients with T2DM were included in this study, consisting of 10 men and 9 women. The age of the patients at the first visit was 69.3 ± 7.3 (59–83) years. The patient’s body mass index was 24.7 ± 4.0 kg/m^2^, and hemoglobin A1c (HbA1c) was 7.5 ± 0.7%.

### 2.2. Ethics Statement

This study was approved by Rakuwakai Research Ethics Review Committee (Approval Number: 01-000137, Approval Date: 28 July 2022). Participants received detailed information about the purpose and an overview of this study before providing written informed consent. The study was conducted according to the Declaration of Helsinki.

### 2.3. Physical Exercise Protocol

All patients received physical therapy (1–2 times per week) at the medical fitness center attached to the rehabilitation clinic, and they performed independent training at home, with instructions to exercise three times per week.

The description of the slow exercise at our institution was as follows: In the slow exercise, lower limb-focused training was performed, which included in-bed exercises around hip joints, bodyweight training (squats, calf raises, and lunges), and machine-based training (leg extension and leg press). The training involved a load of approximately 30–50% 1RM. One set was composed of 3 s of concentric contraction, 1 s of isometric hold, and 3 s of eccentric contraction performed with slow movement [23,24], and repeated 10–15 times. For each exercise, two to three sets of this slow exercise were performed. Before the slow exercise, patients warmed up by aerobic exercise on a treadmill or ergometer for 5–10 min.

As a home exercise, the patients were instructed to perform slow exercises, including bodyweight training (squats, calf raises, and lunges) for 20–30 min per day.

### 2.4. Clinical Evaluations

Hemoglobin A1c (HbA1c) levels were assessed regularly at the outpatient diabetes clinic. Body weight was also measured.

To assess LS, all LS risk tests, including the two-step test, the stand-up test, and the 25-question Geriatric Locomotive Function Scale (GLFS-25), were performed as previously reported [3]. The final LS stage was determined based on the most severe result among all LS risk tests. Subsequently, LS was categorized by severity into non-LS, LS stages 1, 2, and 3 [26]. The score of the two-step test was calculated as the ratio of the maximum stride length of two steps to the individual’s height. Scores of less than 0.9, less than 1.1, less than 1.3, and greater than 1.3 correspond to LS-3, LS-2, LS-1, and non-LS, respectively [27]. The stand-up test was scored on a scale from 0 to 8, with the scores defined as follows: 0 (unable to stand); 1 to 4 (able to stand using both legs from heights of 40, 30, 20, and 10 cm, respectively); and 5 to 8 (able to stand using one leg from heights of 40, 30, 20, and 10 cm, respectively). The scores from 0 to 1, 2, from 3 to 4, and from 5 to 8 points were defined as LS-3, LS-2, LS-1, and non-LS, respectively [27]. The GLFS-25 consists of 25 items rated on a 5-point scale (0–4), yielding a total score ranging from 0 to 100. A higher score indicates greater severity. The domains covered by this scale include body pain (items from 1 to 4), movement-related difficulty (items 5 to 7), usual care (items 8 to 11 and 14), social activities (items 12, 13, and from 15 to 23), and cognition (items 24 and 25) [28]. Total scores from 0 to 6, from 7 to 15, from 16 to 23, and from 24 to 100 are defined to reflect non-LS, LS-1, LS-2, and LS-3, respectively [27].

The five-time sit-to-stand test and four-meter gait speed test were used to assess lower limb function. Grip strength was measured. Both skeletal muscle mass index (SMI) and phase angle (PhA) were measured using a Tanita MC-780 body composition analyzer (Tanita Corporation, Tokyo, Japan). SMI was obtained by normalizing appendicular skeletal muscle mass to height squared. In this study, SMI was used as an indicator of skeletal muscle mass. To assess the upper extremities and lower limbs separately, the skeletal muscle mass of the upper extremities and lower limbs on both sides was divided by squared height, yielding the upper extremity SMI (U-SMI) and lower limb SMI (L-SMI), respectively. When alternating current flows through the body, healthy cell membranes act as capacitors, delaying current flow. This delay creates a phase difference between current and voltage, expressed as the PhA [29]. PhA is recognized as a useful indicator of muscle quality [30]. Left leg PhA was measured as a parameter of muscle quality in this study. The above measurements were conducted at pre-exercise, as well as at 3 and 5 months after the initiation of the exercise program.

### 2.5. Statistical Analysis

Statistical analysis was performed using EZR software (version 1.68, Saitama Medical Center, Jichi Medical University; Saitama, Japan) [31], and results were presented as the mean ± standard deviation (SD). Repeated one-way analysis of variance (ANOVA) was used to evaluate statistical differences among groups (pre-exercise, 3-month, and 5-month). Tukey’s post hoc test was used to determine the specific differences between the groups if the results were significant. Cochran’s Q test was used to compare the distribution of LS stages among groups. The paired *t*-test was used to evaluate the difference in HbA1c between the pre-exercise and 5-month. Differences were considered statistically significant at *p* < 0.05. Graphical representations of the results were generated using GraphPad Prism software (version 10.1.1, GraphPad Software, San Diego, CA, USA).

## 3. Results

### 3.1. Change in HbA1c and Body Weight

HbA1c significantly improved from 7.5 ± 0.7% at pre-exercise to 7.2 ± 0.8% after the 5-month slow exercise protocol (*p* < 0.05). Body weight at pre-exercise, 3 months, and 5 months after exercise initiation was 63.5 ± 14.5 kg, 63.4 ± 14.2 kg, and 63.0 ± 14.6 kg, respectively, with no significant changes over the 5 months.

### 3.2. Change in LS Severity and Improvement in LS Risk Test Outcomes

**Figure 1:**
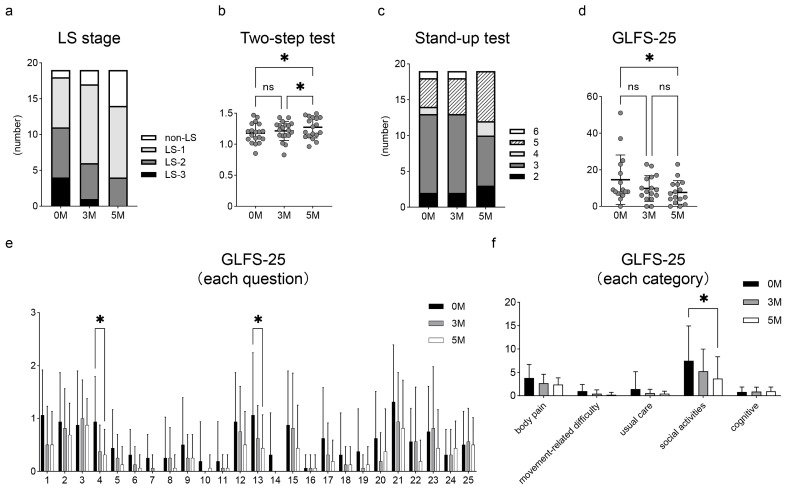
Analysis of LS. Change in (**a**) the distribution of LS stage, (**b**) the two-step test, (**c**) the stand-up test, and (**d**–**f**) GLFS-25. The results of the GLFS-25 analysis for each question are shown in (**e**) and for each category in (**f**). Black dots show the data of each participant. Data are expressed as mean ± standard deviation. * *p* < 0.05. ns, no significant difference; LS, locomotive syndrome; GLFS-25, 25-question Geriatric Locomotive Function Scale.

Figure 1a shows the change in the distribution of LS severities after the initiation of exercise. Cochran’s Q test revealed a statistically significant decrease in LS stage 3 cases (from 4 to 1 to 0) and an increase in non-LS cases (from 1 to 2 to 5) over 5 months. In the LS risk tests, the two-step test scores were 1.17 ± 0.15 at pre-exercise, 1.21 ± 0.15 at 3 months, and 1.27 ± 0.17 at 5 months (Figure 1b). Significant improvements were observed between the pre-exercise and 5 months, and between 3 and 5 months (each *p* < 0.05). The number of participants classified as non-LS (scoring 5–8 points) in the stand-up test was 5, 6, and 7 at pre-exercise, 3 months, and 5 months, respectively (Figure 1c). Regarding the GLFS-25, total scores decreased from 14.6 ± 13.1 at pre-exercise to 9.8 ± 6.8 at 3 months and 7.6 ± 6.3 at 5 months, with a significant reduction between pre-exercise and 5 months (*p* < 0.05) (Figure 1d). When evaluated using each question of the GLFS-25, significant improvements were seen in Question 4 (“To what extent has it been painful to move your body in daily life?”), which relates to body pain, and Question 13 (“To what extent has it been difficult to walk briskly?”), which relates to social activities (each *p* < 0.05) (Figure 1e). Furthermore, when the GLFS-25 was categorized into five domains (body pain, movement-related difficulty, usual care, social activities, and cognitive), the score for social activities significantly decreased over 5 months, from 7.5 ± 7.2 at pre-exercise to 3.7 ± 4.5 at 5 months (*p* < 0.05) (Figure 1f).

### 3.3. Changes in Lower Limb Function Indicators

**Figure 2:**
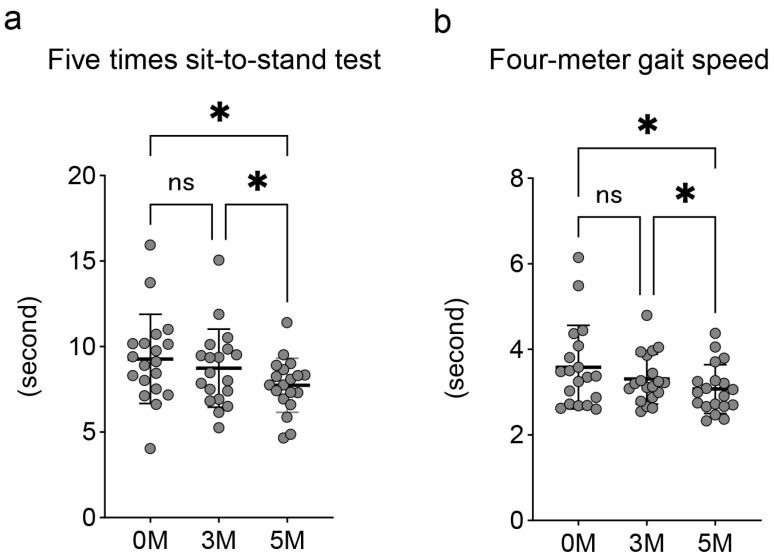
Change in (**a**) the five-time sit-to-stand test and (**b**) four-meter gait speed. Black dots show the data of each participant. Data are expressed as mean ± standard deviation. * *p* < 0.05. ns, no significant difference.

Regarding lower limb function, the five-time sit-to-stand test and four-meter gait test improved significantly at 5 months (7.73 ± 1.54 s and 3.07 ± 0.55 s, respectively) compared with both pre-exercise (9.28 ± 2.53 s and 3.58 ± 0.95 s) and 3 months (8.74 ± 2.22 s and 3.31 ± 0.57 s) (*p* < 0.05 for all) (Figure 2).

### 3.4. Changes in Grip Strength and Skeletal Muscle Indicators

**Figure 3:**
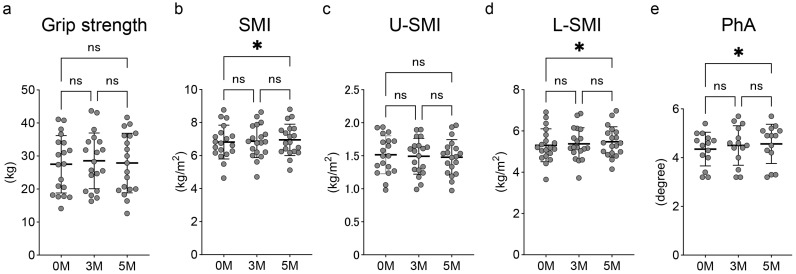
Change in (**a**) grip strength, (**b**) SMI, (**c**) U-SMI, (**d**) L-SMI, and (**e**) PhA. Black dots show the data of each participant. Data are expressed as mean ± standard deviation. * *p* < 0.05. ns, no significant difference; SMI, skeletal muscle mass index; PhA, phase angle; U, upper extremity; L, lower limb.

Grip strength showed no significant change over the 5 months. The values were 27.5 ± 8.4 kg at pre-exercise, 28.6 ± 8.2 kg at 3 months, and 27.9 ± 8.7 kg at 5 months (Figure 3a). The SMI at 5 months (6.95 ± 0.92 kg/m^2^) showed a significant improvement compared to pre-exercise (6.82 ± 1.00 kg/m^2^, *p* < 0.05) (Figure 3b). While the U-SMI did not improve over the 5 months (Figure 3c), the L-SMI at 5 months (5.47 ± 0.71 kg/m^2^) showed a significant increase compared to pre-exercise (5.31 ± 0.78 kg/m^2^, *p* < 0.05) (Figure 3d). The PhA significantly improved at 5 months (4.56 ± 0.78 degrees) compared to the pre-exercise level (4.35 ± 0.67 degrees, *p* < 0.05) (Figure 3e).

## 4. Discussion

In this study, a 5-month lower limb-focused slow exercise program in patients with T2DM led to significant improvements in LS indicators, including improved two-step test performance and reduced GLFS-25 scores. Importantly, these changes were supported by improvements in SMI, PhA, and lower limb function. As a result, the proportion of participants classified as non-LS increased, while those in LS stage 3 decreased, indicating a clinical improvement in lower limb function. Taken together, these findings suggest that a 5-month limb-focused low-intensity exercise not only increases skeletal muscle mass but also improves mobility-related LS outcomes in patients with T2DM.

The exercise therapy is widely recommended for patients with T2DM, not only for improving glycemic control but also for preventing chronic complications such as cardiovascular disease, diabetic neuropathy, nephropathy, and retinopathy [32,33]. However, patients with T2DM often exhibit reduced exercise tolerance [34], which makes it difficult for them to carry out exercise therapy at an appropriate intensity. Therefore, it is crucial to design safe and feasible exercise therapy for this population. Slow exercise, involving low-intensity resistance training with slow movements, could be a safe and effective alternative for patients who cannot tolerate high-intensity exercise due to joint or cardiovascular impairments. In our study, no complications occurred during the 5-month program, supporting its applicability in middle-aged and elderly patients with T2DM. Watanabe et al. reported that even relatively low-intensity (30% 1RM) resistance training with slow movements effectively increased muscle mass and strength in older adults, which was attributed to the sustained skeletal muscle contraction [23]. Physiologically, continuous skeletal muscle contraction of the lower limbs maintains intramuscular pressure at a high level [35], thereby restricting blood flow to the skeletal muscles. Resistance exercise performed under ischemic conditions has been reported to promote skeletal muscle hypertrophy even at low exercise intensity [36]. Consistent with these mechanisms, the slow exercise employed in this study is thought to have contributed to an increase in skeletal muscle mass through a sustained ischemic condition in the muscle. Another study reported that slow exercise not only increases skeletal muscle mass and strength but also significantly reduces HbA1c, thereby improving glycemic control in patients with T2DM [25]. In our study, consistent with the previous report, not only skeletal muscle mass and strength but also HbA1c levels were improved after the 5-month slow exercise program.

LS is defined as a condition characterized by mobility impairment due to decreased lower limb function and is diagnosed using the LS risk tests, which consist of the two-step test, the stand-up test, and the GLFS-25 [26]. Lower limb function is influenced by a variety of musculoskeletal impairments, such as joint pain, restricted joint range of motion, muscle weakness, and balance deficits [37]. Unlike assessments that focus only on skeletal muscle mass or strength, the diagnosis of LS incorporates both functional performance and patient-reported outcomes, enabling a comprehensive evaluation of the effects of exercise therapy. The 5-month lower limb-focused slow exercise program led to progressive improvements in LS severity in this study. This result was supported by the reductions in the five-time sit-to-stand test and the four-meter gait test. This is clinically significant because such improvement was observed even in patients with T2DM, who are prone to skeletal muscle loss. The improvement in LS indicated that slow exercise contributes to reducing fall risk and maintaining independence in patients with T2DM. The two-step test is associated with the maximal gait speed [38], suggesting that the 5-month lower limb-focused slow exercise program improved mobility in patients with T2DM. Moreover, in this study, the improvement in the GLFS-25 social activity domain was especially noteworthy because reduced social activity has been reported to increase the risk of mortality [39].

To the best of our knowledge, this is the first study to evaluate the effectiveness of slow exercise on LS in patients with T2DM. Overall, the present findings offer new insights indicating that slow exercise is a safe, feasible, and effective intervention for reducing the severity of LS and enhancing mobility in this population. A major advantage of slow exercise is that it can be easily implemented in various clinical and community settings, as it requires no specialized equipment and can be performed anytime and anywhere, even by individuals with low exercise tolerance. Importantly, the improvement in LS observed in this study is clinically significant, as it reflects not only an increase in skeletal muscle mass but also enhancements in lower limb function and patient-reported outcomes. These improvements may, in turn, lead to a better quality of life and help extend healthy life expectancy in patients with T2DM.

This study has several limitations. First, the sample size was relatively small. Nevertheless, significant improvement in LS-related indicators was observed, demonstrating the effectiveness of slow exercise in enhancing mobility among patients with T2DM. Second, this study did not include a control group that performed only conventional exercise. Therefore, future comparative studies with conventional exercise are needed to further clarify the effectiveness and safety of slow exercise. However, the improvement in LS among patients with T2DM, who are prone to progressive muscle degradation, represents a clinically significant finding. Third, the participants had heterogeneous antidiabetic medication regimens, and changes in medication during the study period were not fully monitored. Fourth, psychophysiological responses such as ratings of perceived exertion (RPE), session RPE (sRPE), visual analogue scale (VAS), and the Omni scale were not assessed in this study. In the future, large-scale randomized controlled trials that overcome these limitations are expected to provide stronger evidence regarding the effects of slow exercise in patients with T2DM. Finally, although the present study demonstrated significant improvements in LS and lower-limb function, effect size values were not reported due to the small sample size and the exploratory nature of the study. Future larger-scale studies should calculate and report effect sizes (e.g., Cohen’s d, partial η^2^) to better interpret the magnitude and clinical significance of the findings.

## 5. Conclusions

This study demonstrated that a 5-month lower limb-focused low-intensity resistance exercise using slow movements improved LS in patients with T2DM, accompanied by improvements in SMI, PhA, and lower limb function. Our findings suggest that slow exercise could be a safe and effective intervention for improving LS in this population.

## Data Availability

The data supporting the findings of this study are available from the authors upon reasonable request.

## Abbreviations

The following abbreviations are used in this manuscript:

LS	locomotive syndrome
QOL	quality of life
T2DM	type 2 diabetes mellitus
1RM	one-repetition maximum
HbA1c	hemoglobin A1c
GLFS-25	the 25-question Geriatric Locomotive Function Scale
SMI	skeletal muscle mass index
PhA	phase angle
